# Artificial Intelligence Aided Design of Microtextured Surfaces: Application to Controlling Wettability

**DOI:** 10.3390/nano10112287

**Published:** 2020-11-18

**Authors:** Andrés Díaz Lantada, Francisco Franco-Martínez, Stefan Hengsbach, Florian Rupp, Richard Thelen, Klaus Bade

**Affiliations:** 1 Product Development Laboratory, Mechanical Engineering Department, Universidad Politécnica de Madrid, c/ José Gutiérrez Abascal 2, 28006 Madrid, Spain; francisco.franco@upm.es; 2 Institute of Microstructure Technology (IMT), Karlsruhe Institute of Technology (KIT), Hermann-von-Helmholtz Platz 1, 76344 Eggenstein-Leopoldshafen, Germany; stefan.hengsbach@kit.edu (S.H.); florian.rupp@kit.edu (F.R.); richard.thelen@kit.edu (R.T.); klaus.bade@kit.edu (K.B.)

**Keywords:** microtextured surfaces, biointerfaces, artificial intelligence, surface wettability, tribology, direct laser writing, microfabrication

## Abstract

Artificial intelligence (AI) has emerged as a powerful set of tools for engineering innovative materials. However, the AI-aided design of materials textures has not yet been researched in depth. In order to explore the potentials of AI for discovering innovative biointerfaces and engineering materials surfaces, especially for biomedical applications, this study focuses on the control of wettability through design-controlled hierarchical surfaces, whose design is supported and its performance predicted thanks to adequately structured and trained artificial neural networks (ANN). The authors explain the creation of a comprehensive library of microtextured surfaces with well-known wettability properties. Such a library is processed and employed for the generation and training of artificial neural networks, which can predict the actual wetting performance of new design biointerfaces. The present research demonstrates that AI can importantly support the engineering of innovative hierarchical or multiscale surfaces when complex-to-model properties and phenomena, such as wettability and wetting, are involved.

## 1. Introduction

Materials science and engineering are living through tumultuous and extremely exciting decades, through which the materials discovery and industrial application process have been accelerated, in parallel to relevant improvements in human well-being and to the steady growth of varied scientific-technological fields, like tissue engineering and biofabrication, metamaterials and metasurfaces engineering, design of smart devices and structures, to cite a few. Among recent initiatives to further progress in materials discovery supported by computational methods, the Materials Genome Initiative [1,2,3] stands out for proposing the integration of theory, computation and experimentation and the use of accessible and interchangeable data and formats to support researchers and technicians in developing new materials for industrial applications [4,5]. In Europe, the European Materials Modeling Council presents a “Vision Beyond 2020”, in which data integration and machine learning, together with the establishment of online multi-stakeholder innovation hubs, play a fundamental role in new materials development [6]. In any case, it is clear that materials discovery and design supported by computational tools and AI constitutes a new revolution in materials science and engineering with already highly interesting results, especially as regards the prediction of final properties and performance from the chemical composition [7,8,9].

Considering the state-of-the-art, is it important to note that, in spite of the giant potential of the materials genome initiative and of artificial intelligence applied to materials design and discovery, some fundamental issues linked to materials development have not yet been considered or researched in depth. Questions linked to the AI-aided engineering of materials surfaces and to the optimization of related contact properties and tribological performance, in connection to several mechanical and biomedical engineering challenges, remain unexplored. In fact, materials surface features have a direct influence on properties including friction coefficient [10], wear resistance [11], self-cleaning ability [12,13], biocompatible response [14,15,16], ergonomic performance and esthetic aspect [17], among other fundamental characteristics linked to advanced product development in mechanical and biomedical engineering fields. Therefore, they also play determinant roles in materials selection when pursuing innovative functionalities, which can be based on bioinspired design strategies for promoting biological and biomedical applications.

The authors hypothesize that the previously introduced holistic approaches to accelerated materials development, relying on the intensive use of AI if adequately researched and developed focusing on materials surfaces, can prove highly transformative towards high performing devices in several industries. The biomedical industry can greatly benefit from innovative hierarchical surfaces and biointerfaces capable of controlling cell-material interactions, improving biodevices compatibility and incorporating innovative sensing and mechanotransduction functionalities through AI-aided bioinspired design strategies.

In order to explore and better understand the potentials of AI applied to the discovery of innovative biointerfaces and to the engineering of materials surfaces, especially for biomedical applications, this study focuses on the control of wettability through design-controlled hierarchical surfaces (or microtextured biointerfaces), whose design is supported and its performance predicted thanks to adequately structured and trained artificial neural networks (ANN). Wettability is chosen due to its relevance for functional biomedical (micro-)devices, as further explained.

Surface wettability is an interesting property related to surface free energy and to surface topography or geometric micro-/nanostructure [18,19]. Usually, surface wettability is measured through the water contact angle (CA), which helps to classify surfaces as hydrophobic (CA > 90°) or hydrophilic (CA < 90°). Values of CA close to 0° are representative of superhydrophilic surfaces, while values close to 180° are characteristic of superhydrophobic surfaces. There are two main routes for adjusting the wettability of surfaces: the first focuses on chemical functionalization anchoring appropriate molecules upon flat substrates, the second aims at modifying the shapes or topographies of surfaces. These routes may also be synergically combined. Regarding chemical approaches, the wettability of flat surfaces can be fine-tuned by the formation of a monolayer with appropriate hydrophilic or lipophilic functional groups. For instance, gold surfaces can be modified using thiol [20] or carbene [21] anchors, while hydroxylated surfaces such as silicon oxide, glass, mica, etc. can be modified by conventional siloxane chemistry [22]. Both hydrophilic and hydrophobic biointerfaces are interesting: the former for being usually very adequate for interacting with cells and tissues, hence leading more easily to biocompatible medical devices [23]; the latter for their singular self-cleaning properties and ability to stay dry, which can be applied to the development of easy to clean and sterilize surgical instruments [24], to cite some examples.

Recent research has put forward the potentials of creating hydrophobic and hydrophilic transitions upon the surfaces of biomedical microfluidic systems, capable of controlling fluids upon biointerfaces and hence achieving highly multiplexed systems for a wide set of screening and diagnostic purposes [25,26]. While significant advances in the monolayer stabilization have been achieved, topology modification results in more robust and highly applicable surfaces. The possibility of controlling cell behavior and fate through modifications of surface topography, in connection with wettability properties, has also been studied in detail [27]. These advances would not have been possible without parallel progress in micro- and nanomanufacturing technologies and combinations thereof, which enable the straightforward, rapid prototyping and even mass-production of biomedical (micro-) devices with three-dimensional design-controlled surface topographies, as previous studies from our team have shown [28,29,30].

Although the Cassie–Baxter and Wenzel models can model contact angle under different wetting regimes, it is complex to model the actual performance of a surface a priori. The authors hypothesize that AI can help with predicting the actual behavior of fluids upon biointerfaces. In this study, the authors explain the creation of a comprehensive library of microtextured surfaces with well-known wettability properties. Such a library is processed and employed for the generation and training of artificial neural networks, which can predict the wetting performance of new design biointerfaces. The authors demonstrate that AI can importantly support the engineering of innovative hierarchical or multiscale surfaces when complex-to-model properties and phenomena, such as wettability and wetting, are involved.

## 2. Materials and Methods

### 2.1. Creating a Library of Microtextured Surfaces with Known Wettability Properties

**Figure 1:**
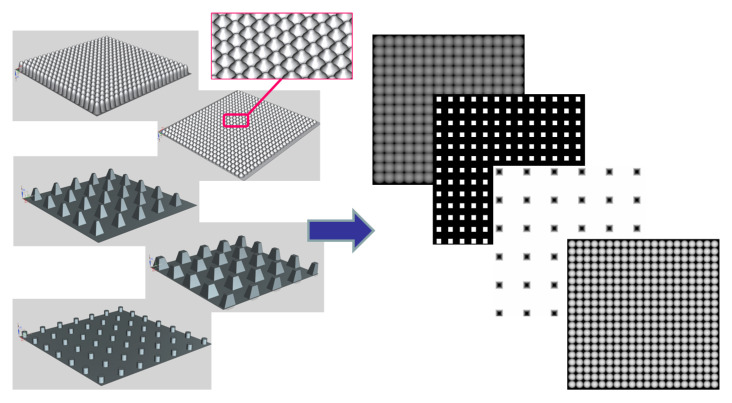
Examples of CAD models of microtextured surfaces (designed with NX), which can be converted into grayscale height maps used for linking the CAD files with matrix-based programming software (MATLAB).

Several studies have dealt with the design and manufacture of microtextured surfaces for controlling the wettability and contact angle of materials surfaces. Both subtractive processes (computer numerical control machining, laser ablation, micro-drilling, etc.) and additive methods (laser stereolithography, digital light processing, powder-based laser fusion, lithography-based ceramic manufacture, etc.) have been applied to the creation of such surface topographies in a wide set of materials. Consequently, there is a plethora of scientific publications, including experiences from our team, describing the wettability properties of different synthetic surfaces. In addition, the epidermis of many living organisms from the animal and vegetal realms have shown very interesting wetting performances, which have also been widely reported. For the research purpose, in order to create a comprehensive library of microtextured surfaces with well-known wettability properties, which will subsequently serve as input for generating and training the artificial neural networks capable of predicting surface contact angle, a selection of publications is gathered. The selection includes relevant research works, in which microtextures are designed and manufactured or directly obtained from nature, with enough information about the surface topographies studied so that they can be replicated and with details about water contact angle obtained through systematic testing [30,31,32,33,34,35]. After selecting the publications, NX 10^®^ (Siemens PLM Software Solutions, Plano, TX, USA) is employed as computer-aided design (CAD) software for modeling the selected microtextured surfaces and completing the CAD library with well-known wettability properties. The CAD models are designed following the descriptions and measurement details included in the consulted references [30,31,32,33,34,35]. In most cases, starting from a planar surface of 1 × 1 mm^2^, the combined use of simple solid-based design tools, like extrusions and revolutions of 2D profiles, and Boolean or pattern-based operations leads to the desired CAD models, as shown in Figure 1 (left). Only for 1 specific case of the collection, which mimics the feature of the lotus plant leaves, Brownian-like microtextures are added to the CAD model in order to achieve truly multiscale or hierarchical surfaces, following previous processes published by our team [30]. In addition, in 4 cases of the collection, due to the extremely fine multiscale details of the CAD model, the starting planar surface measures 0.5 × 0.5 mm^2^ to avoid final CAD files with extremely large sizes (i.e., more than 1 Gb). This does not affect the study, as training of the ANNs is performed with adimensional parameters (see Section 2.3). The CAD files are stored in .stl (standard tessellation language) files for processing.

### 2.2. From 3D CAD Files to Surface Matrices for Further Mathematical Processing

**Figure 2:**
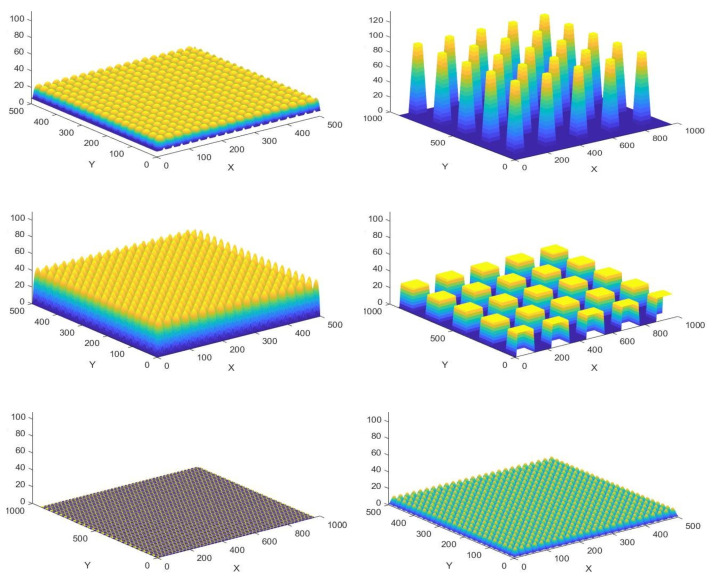
Examples of “MATLAB” microtextured surfaces generated by processing different grayscale height maps.

The extraction of relevant parameters from the microtextured surfaces is performed with the support of matrix-based operations using MATLAB R2020a (The Mathworks, Inc., Natick, MA, USA). The process for straightforwardly transforming the CAD files into MATLAB surface matrices, which store the information of the surfaces, is schematically presented in Figure 1 and Figure 2. An intermediate software is employed for the transformation: Blender, an open-source tool capable of processing and rendering .stl files. Using Blender, each CAD file is viewed using a zenith perspective and stored as grayscale.png images or heightmaps, as shown in Figure 1 (right). Lighter regions correspond to higher values of z, and darker regions correspond to lower values of z.

The .png files are then directly imported with MATLAB, and linear scaling is applied for each of the height matrices obtained so that the absolute height of the microfeatures correspond to the actual dimensions described in the original references. By means of example, Figure 2 presents some of the MATLAB surfaces generated by processing different grayscale height maps, which replicate the features of the original CAD files designed according to references [30,31,32,33,34,35]. Having the surfaces stored in the form of height matrices proves more versatile and direct to process than when employing other CAD import features available in MATLAB, which typically work with .stl files and with their inefficient information storage structures.

### 2.3. Structuring and Training Artificial Neural Networks for Predicting the Wettability of Surfaces

Counting with the surfaces stored in the form of MATLAB surfaces, MATLAB’s neural network Toolbox is employed, as an interesting resource for the direct generation of artificial neural networks, in order to develop a computational model capable of predicting contact angle upon microtextured surfaces.

Two fundamental ratios are used as inputs for training, validating and testing the artificial neural networks. Both are established based on the literature and share some interesting singularities: first, the ratios enable the use of different surfaces without considering the global size, as they are nondimensional; second, both ratios are calculated in a very direct manner, as they only depend on surfaces geometries; and, finally, in a way, they capture the complete essence of the surfaces.

The first ratio employed as input is the “roughness ratio” (R.R), which is used in the Wenzel and Cassie–Baxter models, and it is expressed by the following equation:

$$R.R=\;\frac{real\;surface^{\prime }s\;area}{apparent\;or\;projected\;surface^{\prime }s\;area}=\frac{S_{0}}{S}$$

The second input ratio is the “filled volume ratio” (V.R), which determines the volume filled by the rough surface in a hypothetical prism, which contains the real surface. The prism is defined by the length, height and width of the surface:

$$V.R=\;\frac{volume\;filled\;under\;the\;surface}{volume\;of\;the\;hipothetical\;circumscribed\;prism}=\frac{V_{0}}{V}$$

The filled volume ratio is somehow related to the solid’s area fraction of the Cassie–Baxter’s model, although it is easier to obtain, as it does not depend on the liquid and is only influenced by the topography of the surface. It is linked to the importance of air trapped, within the microtextures, in a heterogeneous wetting state.

The only output used for training, validating, and testing the artificial neural networks is the contact angle. Considering that the library of microtextured surfaces with known wettability properties is developed using the information from a wide set of available studies, which focused on different materials, it is important to minimize the effect of the different materials on wettability and to focus mainly on the microtexture impact on contact angle. Consequently, an incremental contact angle, “ΔCA” (°), is used. It can be defined as: “the contact angle measured upon a microtextured surface minus the contact angle measured upon a planar reference surface of the same material”.

Table 1 presents a summary of the surfaces from the generated library in the form of heightmaps (see Section 2.1 and Section 2.2) and includes, for each surface, the parameters used for training, validating, and testing the ANNs. Enlarged views of the images from Table 1 are included in the “Supplementary Materials 1” section (Table S1), for providing and additional level of detail. Considering that the same topographies applied to planar CAD files of different thicknesses provide the same contact angle values, the library is expanded in a direct way, just by applying the collection of topographies upon planar CAD surfaces with thicknesses of 10 and 20 microns. Therefore, instead of the 23 samples of Table 1, we use a duplicated set of 46 samples for the training, validation, and testing.

Typical ranges of percentages used for data allocation to the training, validation and testing phases are 70–90%, 20–25% and 10–15%, respectively. As no golden rule helps to establish the correct number of neurons and percentages for training, testing and validation, an iterative control process is applied, using the aforementioned ranges and testing combinations of structures between 2 and 20 neurons for the hidden layer. 

The structure of the employed ANNs is based on the two described inputs (surface and volume ratios), the hidden layer with 2 to 20 neurons, an output layer, and the final output value (incremental contact angle). The toolbox automatically splits the values, and the Levenberg–Marquardt method is employed for the training, with the mean square error (MSE) as a loss function. In short, the Levenberg–Marquardt algorithm uses an alternative form of the square descend gradient (SGD) process to optimize the time consumption, as it is possible to perform it without computing the Hessian matrix. Additional details can be found in the MATLAB neuronal networks user guide [36] and a selection of implemented ANNs is to be found in the “Supplementary Materials 2” section for repeatability purposes.

**Figure 3:**
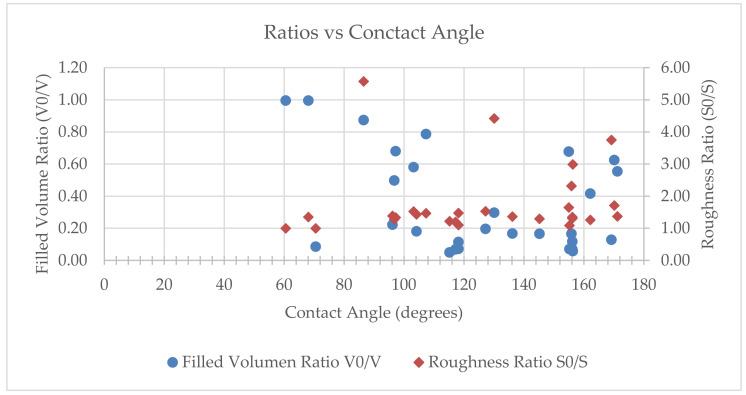

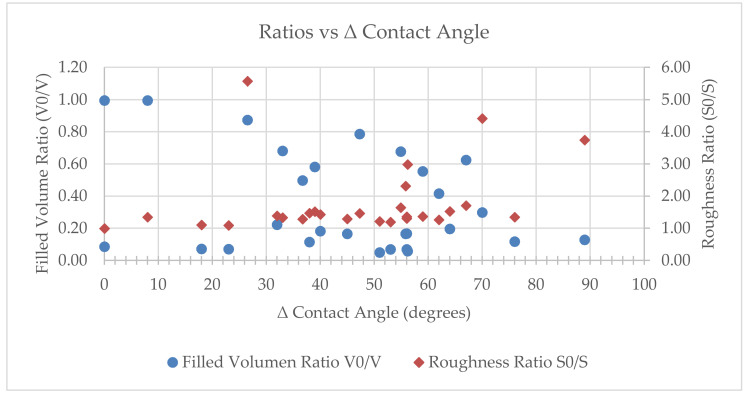
Graphical representations of the roughness ratios (S_0_/S) and filled volume ratios (V_0_/V) of the surfaces from the obtained library, summarized in Table 1, showing highly nonlinear relationships with contact angle (upper image) and incremental contact angle (lower image).

The interest of using AI methods for supporting the engineering of innovative surfaces with desired wettability can be better understood and discussed after a detailed inspection of the data from Table 1. Figure 3 presents graphical representations of the roughness ratios and filled volume ratios of the surfaces from the obtained library. These representations show highly nonlinear relationships between these ratios and the contact angles, both in absolute and incremental forms. In consequence, finding a trend for estimating the wettability of new design surfaces is challenging and can benefit from the use of well-trained artificial neural networks. It is also important to note that existing analytical models do not provide a perfect description for predicting the wettability of innovative biointerfaces directly from design inspection.

Among the many parameters that could have been chosen for describing the surfaces, authors opt for the mentioned roughness and filled volume ratios for different reasons: Firstly, both ratios are deeply connected to Wenzel and Caxie–Baxter seminal works in the field of surface wetting and tribology. Secondly, they are easily computable and are univocally defined, as compared with other possible interesting surface descriptors, like roughness or fractal dimension, which can be defined in different ways and may be affected by the computational process employed to calculate them (i.e., measurement or calculation directions, dimensional range considered). 

Being true that supplementary inputs could have been selected and used for training the artificial neural networks, authors decide to start with a simple artificial neural network structure, also considering the limited number of data available, which in the end proves an adequate decision for this initial study, according to the obtained results (please see Section 3). 

Future updates to the library of materials surfaces will help to increase the number of data and to include additional inputs or outputs, to reach a sort of “super surface classifier”. Even the whole geometry of the surfaces (i.e., the actual.png images) could be used as inputs if other more complex structures like convolutional neural networks were employed, although, in computational terms, this would be much more demanding.

### 2.4. Applying Artificial Intelligence to the Design of Surfaces with Controlled Wettability

#### 2.4.1. Design of Innovative Microtextured Surfaces for Validating the Global Strategy

To evaluate the actual performance and potential real-life applications of the artificial neural networks, as a computational resource for supporting the artificial intelligence-aided design of microtextured surfaces with desired wettability properties, it is necessary to (1) design novel topographies different from those already available in the training library; (2) obtain their characteristic parameters and use them as input for the artificial neural networks, so as to predict a contact angle linked to their wettability; (3) manufacture such novel topographies and assess their actual contact angle values and (4) compare the virtual predictions with the physical measurements.

In consequence, 5 different innovative microtextured surfaces, with potential applications as biointerfaces for several medical devices and bio-MEMS (as detailed in Section 4), are designed following the processes described in Section 2.1 and Section 2.2 with some modifications. In short, we opt for hybridizations and linear combinations among surfaces from the CAD library, towards truly multiscale hierarchical surfaces, whose designs are presented in the results section.

#### 2.4.2. Manufacturing Prototypes of Innovative Microtextured Surfaces for Physical Testing

The manufacturing of the innovative microtextured surfaces prototypes for wettability testing is done using 3D direct laser writing (3D-DLW), also called 3D laser lithography, a high precision AMT based on two-photon polymerization with ultrashort laser pulses, employing the Photonic Professional System from Nanoscribe GmbH (Karlsruhe, Germany).

MATLAB (MathWorks, Inc., Natick, MA, USA) is again employed to generate both the layout data and the data input files (in.stl format) that could be read directly by the Nanoscribe conversion software Describe from Nanoscribe. The Nanoscribe system uses a laser from Toptica (Femto Fiber pro NIR, Munich, Germany) with a wavelength of 780 nm. The setup includes a laser combined with an inverted microscope, which was synchronized and controlled by a PC. The beam is guided through an oil-immersion microscope objective (Zeiss, 63X, NA 1.4, Carl Zeiss AG, Oberkochen, Germany) and focuses on a resist (acrylate-based Ip-DIP, Nanoscribe), previously placed upon a glass substrate rinsed with 2-propanol. For better adhesion of the written geometries, the substrate is usually heated to 120 °C for 10 min. The mounted glass substrate is moved by motor stages (Physics Instruments M511.HD1, Physik Instrumente GmbH and Co. KG, Karlsruhe Germany), and a piezoelectric driver (Physics Instruments P-562.3CD, Physik Instrumente GmbH and Co. KG, Karlsruhe Germany) is used for z-travel.

The technology had been used in previous research by our team [28,29,30]. Here, we applied it to write larger fields with high precision and create prototypes of innovative “AI-aided” designs, which enabled performing the wettability tests needed for analyzing the prediction potential of the generated and trained artificial neural networks.

For this study, the structures are created by writing tiles (300 µm × 300 µm) with feature sizes of 1 µm. To resolve the feature sizes, the structures are converted for writing with a resolution of 0.35 µm in the z-direction (slicing) and 0.25 µm in the x–y direction (hatching). To archive this resolution, the small configuration set (SF-set) from Nanoscribe is employed. This setting contains the usage of a 63x objective (Zeiss, 63X, NA 1.4, Carl Zeiss AG, Oberkochen, Germany), IP-Dip as photoresist (IP-Di, Nanoscribe GmbH) and fused silica (25 mm × 25 mm × 0.7 mm) as substrate. Since the specified writing field of this configuration is 140 µm × 140 µm, the design is split into tiles of that size. To archive larger areas, the tiles are stitched together. In this case, stitching is applied to a size of 1.8 mm × 1.8 mm. Before the DLW process, the fused silica substrate is rinsed with 2-propanol and acetone, followed by a dehydration step for better adhesion. This is done on a hotplate at 120 °C for 10 min. For drying the DL-written samples, a critical point dryer (Automegasamdri^®^-915b, Tousimis, Rockville, MD, USA) is employed.

Once the prototypes of the microtextures surfaces are manufactured, scanning electron microscopy (SEM) imaging is also used for visualization purposes. An SEM system by Carl Zeiss AG (Oberkochen, Germany) is employed.

#### 2.4.3. Wettability Testing and Imaging Procedures and Resources

Wettability testing of the microtextured surface prototypes is necessary to understand and verify the predictive potential of the generated and trained artificial neural networks when applied to forecasting the contact angle upon innovative textures. For such purpose, an experimental setup with a micro-droplet generator, a precision measuring stage, and a high-resolution optical camera with extra lighting is used. Initial tests are performed upon a planar surface manufactured using IP-Dip for obtaining a reference value for a contact angle of 60 ± 1º. Subsequently, two measurements are carried out upon each of the 1.8 × 1.8 mm^2^ manufactured microtextured surfaces. Water droplets of 2 μL are employed. A laboratory with a monitored environment is used: a temperature of 21.5 ± 0.5 °C and relative humidity of 37 ± 2% are monitored as working conditions during measurements.

## 3. Results and Discussion

### 3.1. CAD Models, Prototypes and Wetting Response of the Innovative Microtextured Surfaces

The gathered collection of microtextured surfaces is a starting point aimed at creating a most comprehensive library of surface topographies with information about their wetting response, which can be continuously updated. Such updates can be used for further training the ANNs, once additional testing results upon physical surfaces are available. The library already includes several CAD files in .prt and .stl formats, as well as their equivalent topographic maps stored in the form of matrices and is available for researchers in the field wishing to collaborate or test alternative approaches linked to the AI-aided design of textured biointerfaces.

**Figure 4:**
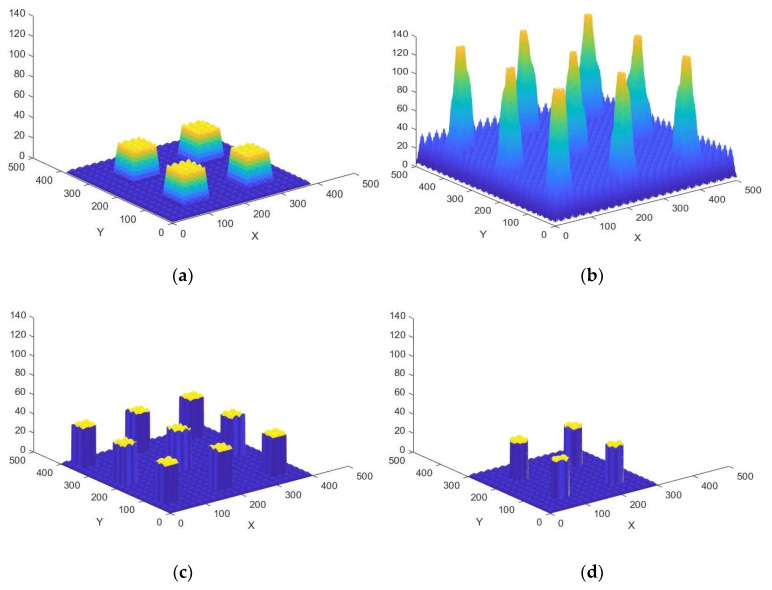

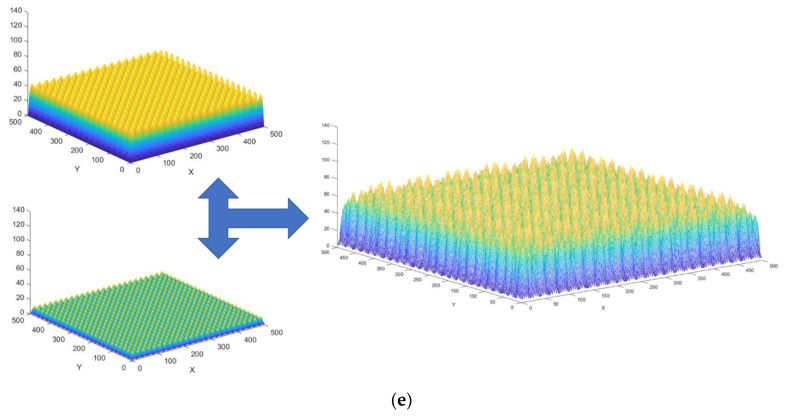
(**a**–**e**): Different microtextured, multiscale or hierarchical biointerfaces designed for being manufactured and tested to analyze the prediction quality of the ANNs developed and validate the global strategy. Dimensions shown in axes in μm.

**Figure 5:**
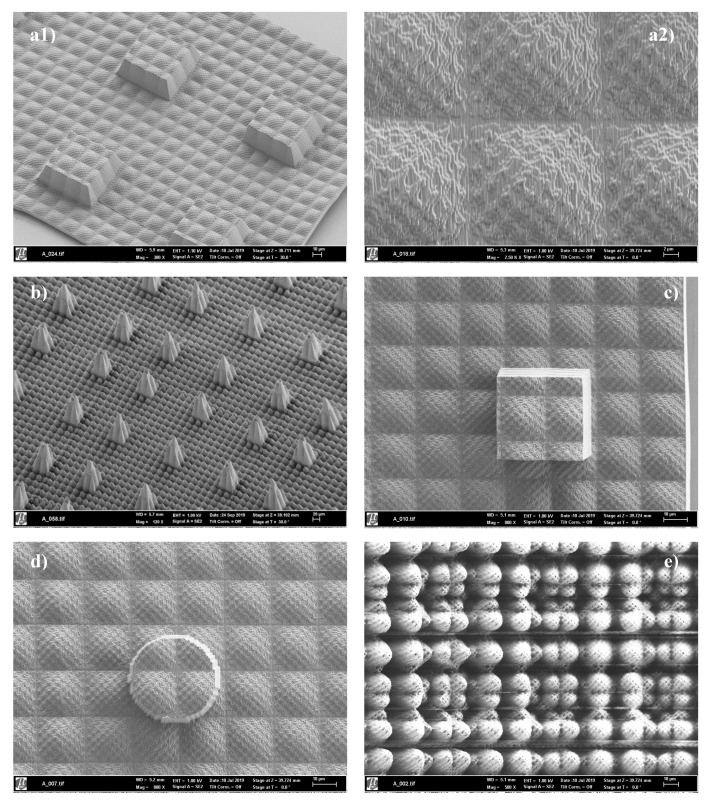
(**a**–**e**): Scanning electron microscopy images from the different direct laser written microtextured biointerfaces, obtained in photopolymerizable resin after the multiscale or hierarchical designs presented in Figure 4. Images a–e from Figure 5 correspond to the designs a–e from Figure 4.

Regarding the five newly designed microtextured surfaces or biointerfaces, envisioned for being manufactured and tested, in order to analyze the prediction quality of the ANNs developed, Figure 4 presents the design results (designs, a, b, c, d, e) and Figure 5 show their prototyping by direct laser writing. The different designs include hybridizations or combinations among existing surfaces from the collection, after performing scaling in the different x, y, z directions and focusing on the creation of truly hierarchical or multiscale topographies. 

To this end, four designs (Figure 4a–d) are achieved by adding biomimetic microtextured bumps to periodic arrays of different types of pyramids, prisms and cylinders, following a design process developed by our team and previously explained [30]. While the periodic pyramids, prisms and cylinders are features in the 30–100 μm height range, the microtextured bumps include wavy features with an amplitude of nearly 5 μm and additional random features with an amplitude of around 1 μm, all of which leads to very representative multiscale hierarchical topographies. 

Such hierarchical surfaces are well-known for their potential hydrophobicity and are characteristic of the epidermis of several plants. An additional design is achieved by combining two biomimetic surfaces from the library (Figure 4e), one with the topography of the lotus plant leaves, one with the topography of the viola flower leaves, both hydrophobic. The rationale behind is trying to combine two already hydrophobic biointerfaces from the natural realm and analyzing if the final multiscale combination leads to an improved result in terms of self-cleaning properties., Authors expected a very hydrophobic response from such a nature-inspired design, as also happened with the prediction performed by the ANNs, as discussed in Section 3.2.

To our knowledge, the presented designs provide new examples of hierarchical biointerfaces, counting with simple periodic features combined with wavy and random functions, at least one order of magnitude smaller than the simple periodic features and almost reaching the nanometric range. In a way, they enter the realm of metasurfaces and, apart from the proposed application, linked to controlled wettability, similar hierarchical surfaces may have varied applications, both as biointerfaces for interacting at cellular level, but also in connection to basic research studies in acoustics and electromagnetism, among other fields.

Again, it is important to point out the interest of counting with a collection of microtextured interfaces, stored in the form of matrices: the near direct application of linear transformations and combinations among the surfaces from the collection can help to rapidly increase the number of samples in the library, even in an automated way and bridging the gap across different scales (nano-micro-meso-macro). Counting with AI tools capable of analyzing the new designs may prove a powerful tool for the AI-assisted discovery of biointerfaces with interesting features and responses for many different fields of study and potential industrial application, even beyond those linked to the biomedical field.

Taking manufacturing results into account, which are shown in Figure 5, it is relevant to highlight the outstanding accuracy of the additive manufacturing technology employed and the almost perfect replication of the designed microtextured biointerfaces. The stitching between periodic regions, performed for achieving larger structured regions, as required for wettability testing, works adequately.

Although in a first attempt, some detachments between the processed resins and the glass substrates appear, these are importantly minimized in a second attempt by applying gentler postprocessing and drying conditions. Still, some minor detachments between resin and glass are present in the outer borders, but authors consider the structured geometries adequate for wettability testing. Figure 5(a2) provides an enlarged view of the microtextured surface of Figure 5(a1) and helps to put forward the precision and quality of the direct laser writing procedure and shows perfectly closed and solid microtextured biointerfaces.

**Figure 6:**
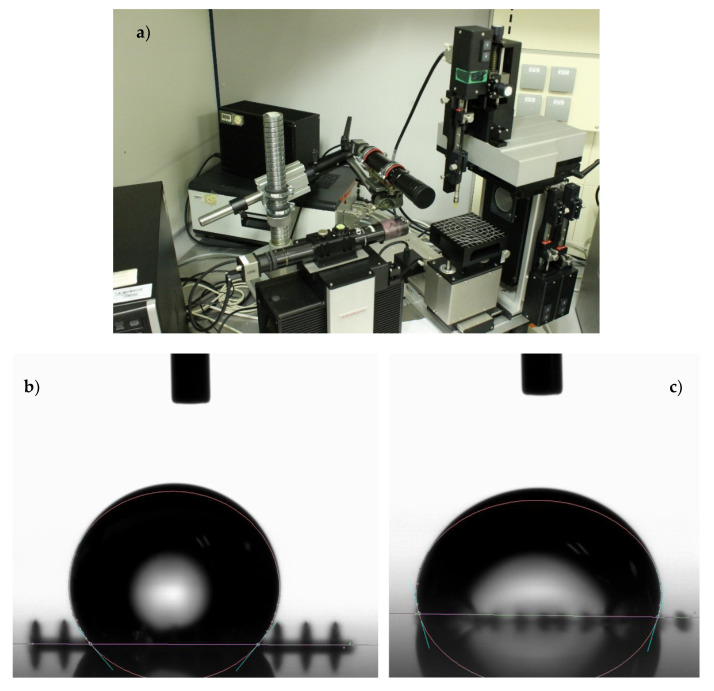
(**a**) Experimental setup for wettability testing. (**b**) Wettability testing result example: droplet upon the design shown in Figure 4b. (**c**) Wettability testing result example: droplet upon the design shown in Figure 4c.

Once the prototypes are obtained by DLW, wettability testing is performed using the setup described in Section 2.4.3 and shown in Figure 6a. By means of example, two results of the different tests are presented in Figure 6b,c, which respectively provide information about the contact angle for designs presented in Figure 4c,d. These experimental results are fundamental for validation purposes and for analyzing the potentials of AI for supporting the optimization of surface textures when complex phenomena like wettability are involved, as discussed in the following subsection.

### 3.2. Performance of the Structured and Trained Artificial Neural Networks: Predictions vs. Real Performance

In general, the training method uses the back-propagation algorithm, and the loss function is the mean square error. Considering the essentials of the back-propagation algorithm and the network’s code, it is possible for the network to prioritize one of the features, in this case, one of the ratios. In addition, these types of algorithms may be affected by the scale of the input. Summarizing, if the two ratios are not on the same scale or are not standardized, the training process may perform worse because they may prioritize the input with larger values. Therefore, it is important to further explain and discuss MATLAB’s neuronal network code: The toolbox includes an algorithm to rescale the dataset between −1 and 1 by applying the “mapminmax” function to the inputs and outputs by default, which helps to improve the training process in a preprocessing stage. This option is applied in the study as well, both for inputs and outputs or targets.

Once preprocessing is performed, the real neuronal network starts to train, seeking the minimization of the loss function. The type of net function in the hidden layer is the hyperbolic tangent sigmoid function (“tansig”), which proves adequate for this type of problem and for the selected algorithm. Finally, the ANN code includes an output layer with one neuron and a postprocessing step to bring back the targets to the real scale by using the gain and offset to do an inverse rescaled process. The above explanation helps to understand how the artificial neuronal networks work, why they can predict the contact angles in this study and the reason for differences between varied artificial neural networks generated and studied, which differ in the number of neurons and in the splitting between the training, testing and validation datasets.

After the generation process of 267 artificial neuronal networks, with neurons between 2 and 20 and many combinations of sample percentages, for the training, testing and validating phases, the team checks the predictions for the newly designed surfaces and their real wetting performance. Table 2 presents the absolute errors (AE measured in **°**) between the contact angle predictions provided by three selected neural networks and the real measurements performed for the five designed, prototyped, and tested surfaces. These absolute errors, shown in Table 2, follow the same sorted list of the samples from Figure 4, Figure 5 and Figure 6, as also detailed in Table 3, which summarizes the wettability testing results. In other words, AE_1_, AE_2_, AE_3_… are sorted according to the design, manufacturing and wettability testing sequences shown in Figure 4, Figure 5 and Figure 6. The three selected artificial neural networks’ source codes are presented, as MATLAB .m files, in the “Supplementary Materials 2” section, for replicability purposes.

About the quality of the predictions, it is important to remark that in four out of five surfaces, the absolute errors are below 5°, which is remarkable in the opinion of the authors. It is necessary to note that for the 4th surface (shown in Figure 4d and Figure 5d), the wettability test provided an unexpected result, with an incremental contact angle close to 0, possibly due to a design or manufacturing defect, like a problem with the stitching between periodic regions or a detachment between printed material and substrate. Nevertheless, it is important to present a complete overview of the whole experiment. According to the results, neural network 2 from Table 2 provides the most interesting results. The obtained absolute errors are also included in Table 3, together with the images from the designed and manufactured surfaces, with the results from wettability testing and with the surface and volume ratios that characterize such surfaces.

Taking apart the case of design 3d, whose performance is highly hydrophilic, in contrast with the expectable behavior considering related microtextures from the CAD library and available references, the ANNs are able to predict the performance of the innovative biointerfaces quite remarkably. The case of design 3d should be further analyzed, as probably a flaw in the design, manufacturing or testing method, perhaps a lack of polymerization of the whole surface or an inner detachment from the glass substrate, lead to the improbable result. Moreover, apart from the artificial neural networks shown in Table 2, 21 additional ANNs from the collection can estimate the contact angle of the new designs with an absolute error of less than 10° for 4 out of 5 samples.

This performance is even better than that of experienced researchers devoted to the engineering of surfaces for controlling wettability. In fact, the ANNs are able to predict the contact angle values in a much more precise way than the team of authors, after having studied several references and classified the biointerfaces that constitute the CAD and matrices collections used for training. The generalization potential of the ANNs, based on just two input parameters for each sample, is quite noteworthy, considering that the information used as input for the ANNs is extremely synthetic when compared to the whole information of each sample stored as .stl CAD file or as.jpg or.png microtopographic map.

An extremely interesting example that helps to understand the generalization degree achievable by the ANNs is the case of design 3e, which hybridizes the macrobumps of the viola flower petals with the hierarchical microstructures of the lotus plant leaves. The initially expected behavior for this bioinspired example is that of a highly hydrophobic surface. Probably an underlaying human bias leads to the argument that, if two superhydrophobic surfaces are hybridized or combined, the result should be even more hydrophobic or at least highly hydrophobic. However, the generated ANNs, working just with surface and volume ratios, can predict that the combination leading to design 3e is not as hydrophobic as the designers expected. The ANNs generalize that intricate and hierarchical surfaces, characterized by large surface ratios, and that microtextures with large aspect ratios, leading to low volume ratios, combinedly provide the highest contact angles.

In a way, this helps to clearly illustrate the interest of counting with AI supporting tools for the design and in silico evaluation of innovative biointerfaces designs, especially when complex phenomena, such as wetting and tribological issues, are involved. Well trained ANNs may help to evaluate, in a very automated way, thousands of microtextured surfaces for screening purposes before performing a reduced selection of potentially adequate solutions for further manufacturing and testing, hence helping to work more efficiently, sustainably and cost-effectively.

## 4. Challenges and Future Proposals

### 4.1. Potentials, Limitations and Challenges of the Study

AI has intrinsic limits, including the need for large data for achieving desired results, the “black box” problem, issues with overfitting, which may lead to the failure of some of the planned learning strategies. The use of progressive neural networks for multitasking, of multitask learning using uncertainty, of evolution and learning and of generative models, as main alternatives to classical neural networks, towards the generation of surfaces with desired properties, may be strategies to explore in the near future, so as to minimize failure. Some options are proposed below, when dealing with future research directions, to overcome common limitations and challenges usual in the artificial intelligence field. Ideally, the developed AI tools will lead, not just to predicting and classifying, but also a better understanding of the behavior of natural and synthetic surfaces and to more adequate AI-aided processes for the engineering of innovative surfaces. This study has dealt with the prediction of contact angle upon microtextured biointerfaces, which allows classifying into hydrophobic and hydrophilic surfaces, and demonstrated the remarkable interest of ANNs for reaching reliable predictions, at least more reliable than those based on human experience when applied to envisaging the performance of new textures. Potentially, these processes can be applied to the automated discovery of surfaces with desired contact phenomena, especially if some current limitations are solved and if the future research proposals discussed below are considered.

Although a huge number of research studies deal with micro/nanomanufacturing strategies for the production of highly hydrophobic materials, surfaces and biointerfaces, due to their interesting self-cleaning antifouling properties, the number of publications that detail methods based just on surface topography modifications, without the application of chemical functionalizations or thin films, is not so large. In many cases, the information available about the topographies is not enough for performing a CAD model or for obtaining a heightmap. In consequence, a limitation of the presented study is linked to the reduced number of samples within the collection of designs (the 23 interfaces of Table 1), which is developed based on a handful of highly selected references with the information presented in a very clear and systematic way [30,31,32,33,34,35]. Even though the materials and methods from such references are varied, the fact that these studies presented wettability tests upon the materials of interest, both before and after microtexturing, enables working with incremental values of contact angle for constructing the collection, which proves adequate for minimizing the variability of inputs and focusing mainly on the topographical effects. Counting with a larger set of samples for training and increasing the library of microtextured surfaces, with information from additional wetting tests, all of them performed using the same materials and methods, is important for future studies. However, it is important to understand the relevance of the results obtained and the promising generalization power and prediction ability of the ANNs, which have provided accurate predictions about the wetting performance of innovative microtextured interfaces, even outperforming human estimates based on experience.

### 4.2. Future Research Proposals

Considering future research directions and proposals, it is important to put forward the interest of further exploring the applicability of artificial intelligence to predicting the properties of engineered surfaces and to supporting the discovery and design of innovative materials. Interesting alternatives to the use of ANNs with surface and volume ratios as inputs include the employment of convolutional neural networks, using the topographic maps or even microscopic images of the microtextured surfaces as inputs, as well as the utilization of strategies for expanding the available dataset based on teacher-student algorithms. Comparing the overall precision attainable with different approaches, and pondering the computational resources needed, is essential to achieve an optimal method. Better prediction accuracy will also require from the design, manufacture, and evaluation of tens or even hundreds of additional innovative topographies, with which the artificial neural networks will further learn for increased versatility. 

Once an extremely comprehensive collection of surfaces and properties is used for the creation of an AI-based “super-predictor or classifier” of microtexture performance, the automated discovery of innovative surfaces with desired properties will be enabled: surface topographies will be generated in a loop by using mathematical functions, and such topographies will be screened by the predictor or classifier. All this applies to the wetting performance of polymeric biointerfaces attainable by direct laser writing but can be expanded to other materials and properties just by enlarging the set of samples, modifying the inputs and rearranging the training, validation and testing processes.

Apart from the intrinsic interest of the described processes and proposed trends, in connection with the application of AI to materials science and engineering, a wide set of industrial applications based on the AI-assisted design of microtextured interfaces can already be discussed here. Among plans for future research, an outstanding direction is linked to applying these AI-assisted design and manufacturing processes for defining biomimetic transitions of topography and wettability upon biointerfaces in order to control cell behaviors and fate within microfluidic systems for diagnostic and labs-/organs-on-chips for modeling diseases. Studying the effect of AI-designed microtextures on the biocompatibility and long-term integration of implants constitutes another remarkable field of application, especially if the library is further completed with several additional microtextures from biomaterials and biological tissues and if the networks learn from such information. The potential manufacture of AI-based biointerfaces using smart materials, like shape-memory polymer foils, can open new horizons in the area of smart materials and structures; for devices in which the wetting performance may be selectively modified along the life cycle.

## 5. Conclusions

Curiously, the ancient Greek term for “surface”, “επιφάνεια” or “epifáneia”, is polysemic and refers to the visible surface of an object, to arising from something unexpected and to a manifestation, typically a “eureka” moment, corresponding to a new beginning, among other possibilities [37]. In a parallel way, authors consider that the application of artificial intelligence to the design of innovative hierarchical surfaces and micro-/nano-textured biointerfaces may also constitute a sort of daybreak in surface engineering, which can help to rethink several scientific and technological fields, including tribology, ergonomics, esthetics, optics and design, to cite some examples. Further studies linked to the progressive implementation and tuning of computational methods presented and discussed in this study may promote the straightforward, geometrically trustworthy, structurally reliable, resource-efficient design and automated “intelligent” development of synthetic materials surfaces and, hence, accelerate their impact as smart biointerfaces for advanced product design in the biomedical engineering field. Considering the presented results and the analyzed potentials, the AI-aided discovery of biointerfaces can undoubtedly constitute an excellent complement to ongoing research directions and available methods in the area of AI applied to materials sciences and engineering, especially as regards the development of functional engineering materials.

## Figures and Tables

**Table 1:** Summary of surfaces from the generated library and parameters used for training ANNs.

Surface View	Surface	CA (°)	ΔCA (°)	V_0_/V	S_0_/S	Ref.
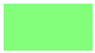	1	≈60	0	0.996004 (1.0)	0.996004 (1.0)	Present study
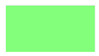	2	≈70	0	0.996004 (1.0)	0.996004 (1.0)	[27]
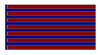	3	96	32	0.681005	1.385686	[28]
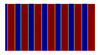	4	97	33	0.581493	1.332758	[28]
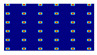	5	103	39	0.050000	1.520136	[28]
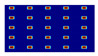	6	104	40	0.06869	1.433589	[28]
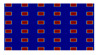	7	115	51	0.196086	1.219188	[28]
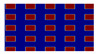	8	117	53	0.297979	1.196545	[28]
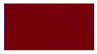	9	118	18	0.874239	1.102110	[29]
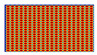	10	118	38	0.787298	1.470737	[30]
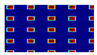	11	127	64	0.117611	1.529714	[28]
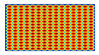	12	136	56	0.677470	1.364492	[30]
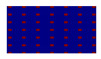	13	145	45	0.072338	1.293813	[29]
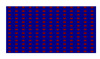	14	154.9	54.9	0.167083	1.645769	[31]
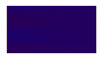	15	155	23	0.222987	1.08823	[29]
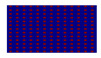	16	155.8	55.8	0.166315	2.317966	[31]
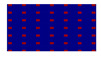	17	156	56	0.069969	1.305968	[29]
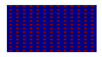	18	156.2	56.2	0.166411	2.987631	[31]
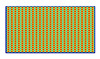	19	156	76	0.624982	1.349546	[30]
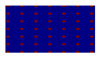	20	162	62	0.057735	1.261909	[32]
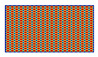	21	169	89	0.554934	3.748147	[30]
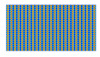	22	170	67	0.415654	1.707897	[27]
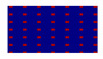	23	171	59	0.069264	1.366480	[29]

**Table 2:** Absolute errors, measured in degrees (°), between the contact angle predictions provided by 3 selected artificial neural networks and the real measurements performed for the 5 surfaces designed, prototyped, and tested.

Neuronal Network	Neurons	AE_1_ (°)	AE_2_ (°)	AE_3_ (°)	AE_4_ (°)	AE_5_ (°)
1	8	3.713	3.074	1.288	36.671	0.268
2	7	0.235	2.295	3.9059	31.927	0.763
3	13	3.207	0.061	1.138	39.448	0.183

**Table 3:** Manufactured surfaces, measurement results (CA_m_ and ΔCA_m_), predictions (CA_p_ and ΔCA_p_), sample ratios and absolute prediction errors for the best performing artificial neural network (ANN2 from Table 2).

Surface View (CAD and Prototype)	CA_m_ (°)	ΔCA_m_ (°)	CA_p_ (°)	ΔCA_p_ (°)	V_0_/V	S_0_/S	AE (°)
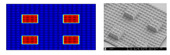	96.7	36.7	96.9352	36.9352	0.1819	1.2823	0.235
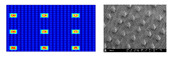	130	70	129.9391	69.9391	0.1290	4.4183	2.295
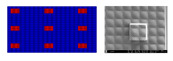	107.3	47.3	106.1618	46.1618	0.1147	1.4630	3.9059
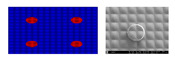	68	8	99.9270	39.9270	0.0856	1.3471	31.927
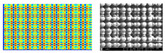	86.5	26.5	86.3132	26.3132	0.4977	5.5729	0.763

## Supplementary Materials

The following are available online at https://www.mdpi.com/2079-4991/10/11/2287/s1, Supplementary Materials 1: Table S1. Enlarged views, in the form of topographic maps, of the microtextured surfaces from the collection (after Table 1); Supplementary Materials 2: Source code (MATLAB’s .m files) for artificial neural networks 1, 2 and 3, whose results of contact angle prediction are shown in Table 2 and Table 3.
