# Supplementary material for: Artificial Intelligence Aided Design of Microtextured Surfaces: Application to Controlling Wettability
Source: Nanomaterials (Basel). 2020 Nov 18;10(11):2287. doi: 10.3390/nano10112287 (PMC7698866; doi:10.3390/nano10112287)
Supplement: Supplementary file 1 [file nanomaterials-10-02287-s001.pdf]

Supplementary Materials:

Artificial Intelligence Aided Design of Microtextured Surfaces: Application to Controlling Wettability

Andrés Díaz Lantada [1,*], Francisco Franco-Martínez [1], Stefan Hengsbach [2], Florian Rupp [2], Richard Thelen [2] and Klaus Bade [2]

[1] Product Development Laboratory, Mechanical Engineering Department, Universidad Politécnica de Madrid; c/ José Gutiérrez Abascal 2, 28006 Madrid, Spain; francisco.franco@upm.es

[2] Institute of Microstructure Technology (IMT), Karlsruhe Institute of Technology (KIT), Hermann-von-Helmholtz Platz 1, 76344 Eggenstein-Leopoldshafen, Germany; stefan.hengsbach@kit.edu (S.H.); florian.rupp@kit.edu (F.R.); richard.thelen@kit.edu (R.T.); klaus.bade@kit.edu (K.B.)

* Correspondence: adiaz@etsii.upm.es

Supplementary Materials 1

Table S1. Enlarged views, in the form of topographic maps, of the microtextured surfaces from the collection (after Table 1).

**Surface View**	
Table S1: 1	Table S1: 2
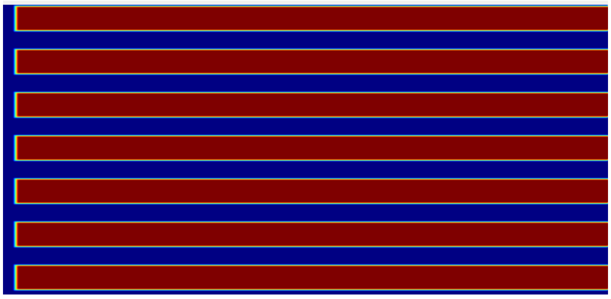 Table S1: 3	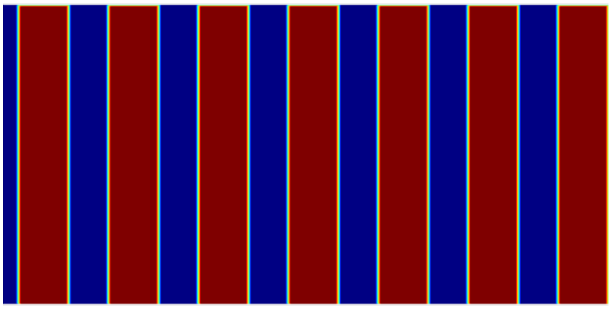 Table S1: 4
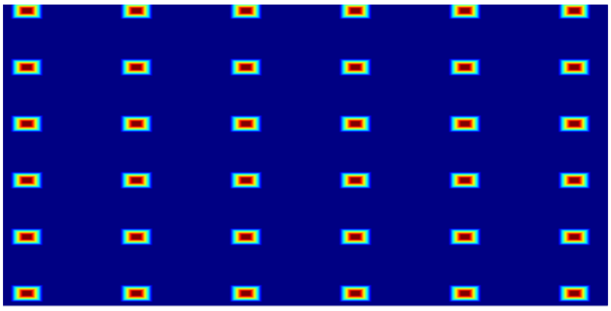 Table S1: 5	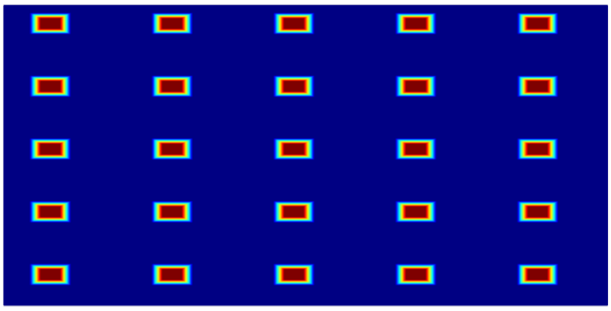 Table S1: 6

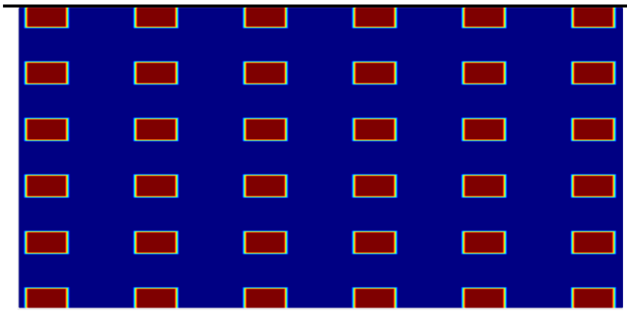
Table S1: 7

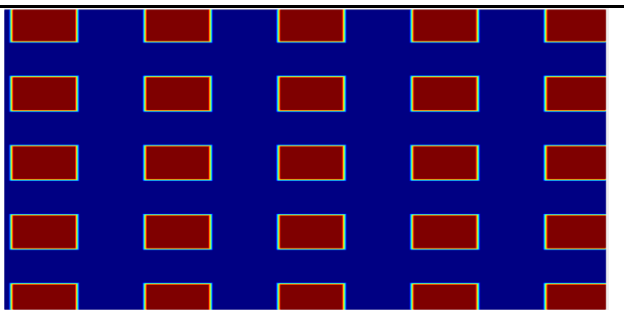
Table S1: 8

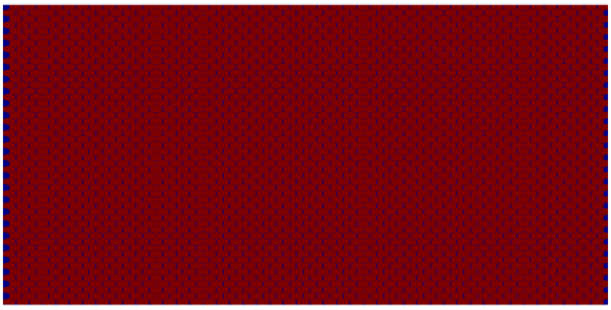
Table S1: 9

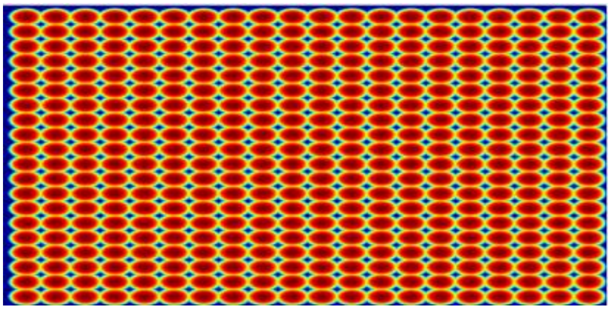
Table S1: 10

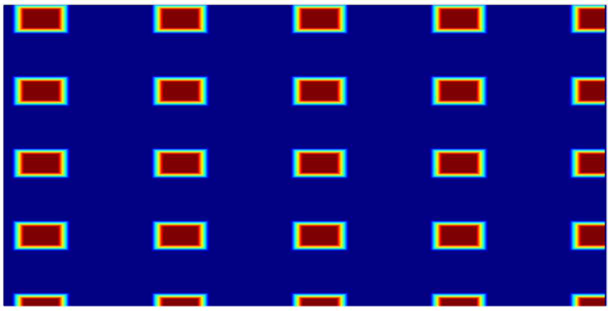
Table S1: 11

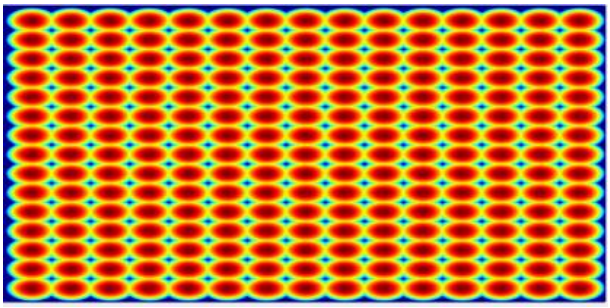
Table: S1: 12

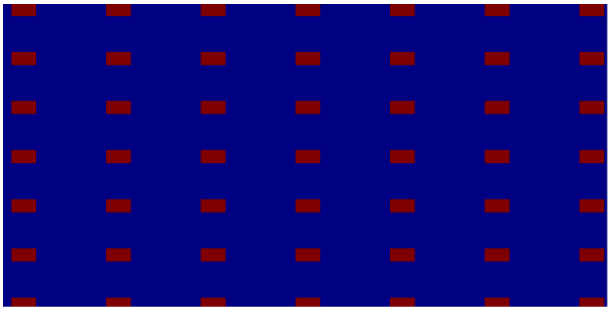
Table S1: 13

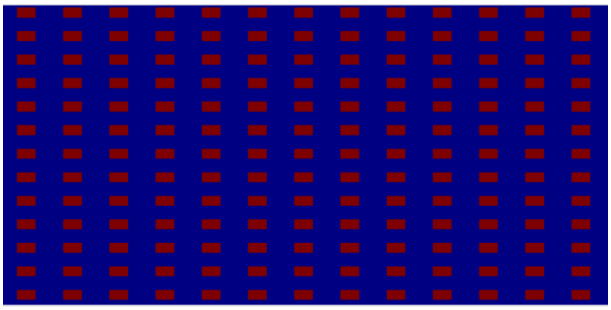
Table S1: 14

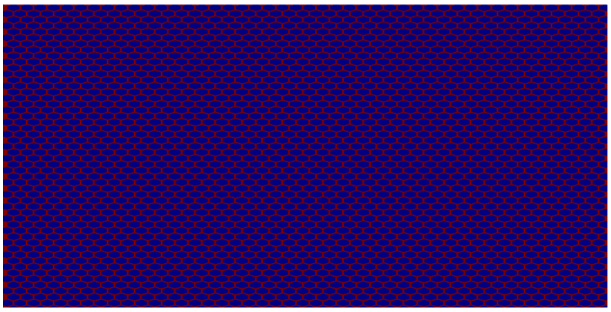
Table S1: 15

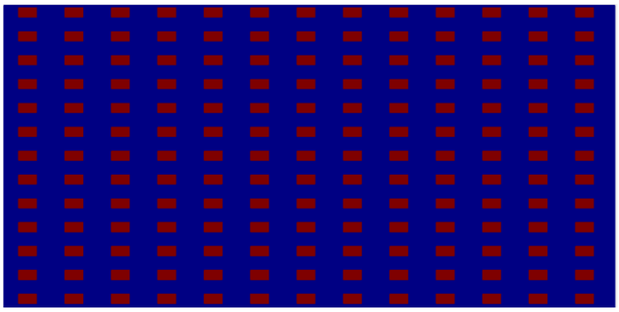
Table S1: 16

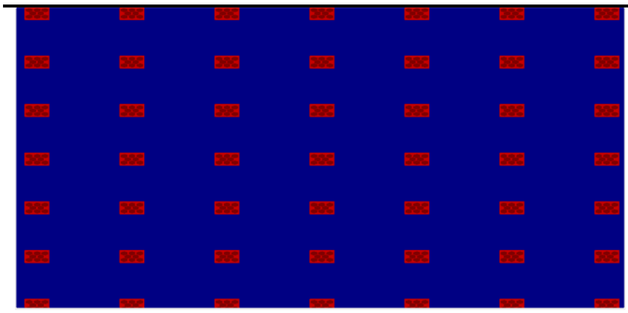

Table S1: 17

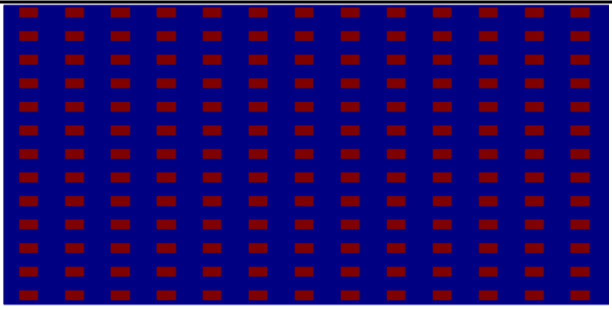

Table S1: 18

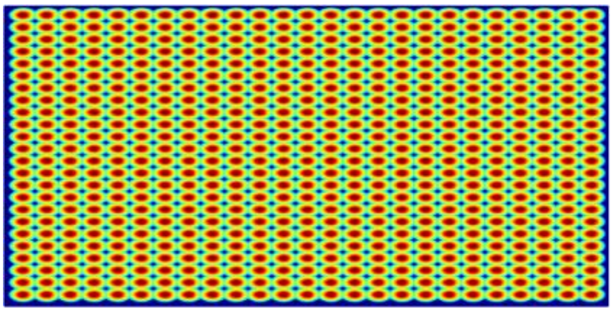

Table S1: 19

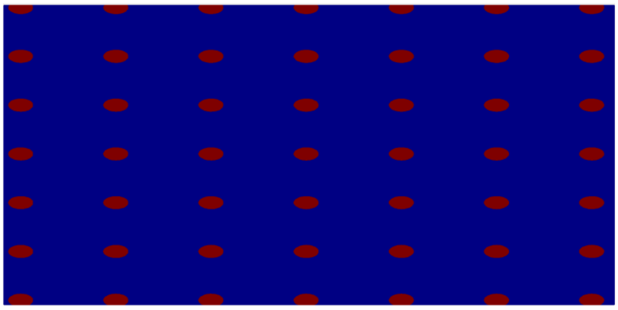

Table S1: 20

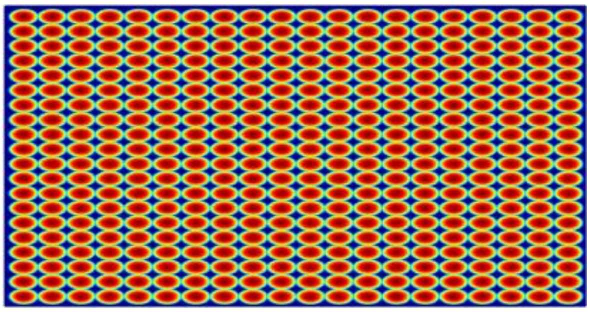

Table S1: 21

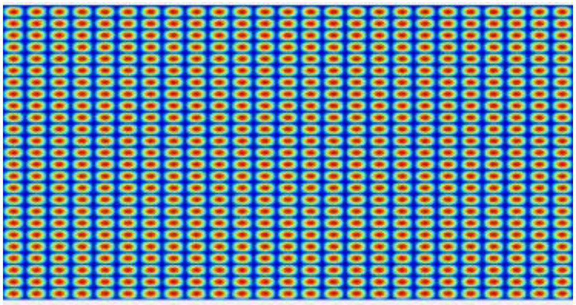

Table S1: 22

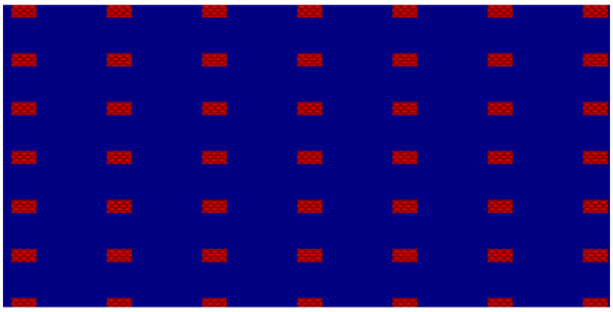

Table S1: 23

Supplementary Materials 2

Source code (MATLAB’s .m files) for artificial neural networks 1, 2 and 3, whose results of contact angle prediction are shown in Tables 2 and 3 Table 2; Table 3.

Artificial neural network 1:

```
function [Y,Xf,Af] = myNeuralNetworkFunction(X,~,~)
%MYNEURALNETWORKFUNCTION neural network simulation function.
%
% Auto-generated by MATLAB, 02-Jan-2020 19:29:38.
%
% [Y] = myNeuralNetworkFunction(X,~,~) takes these arguments:
%
%   X = 1xTS cell, 1 inputs over TS timesteps
%   Each X{1,ts} = Qx2 matrix, input #1 at timestep ts.
%
% and returns:
%   Y = 1xTS cell of 1 outputs over TS timesteps.
%   Each Y{1,ts} = Qx1 matrix, output #1 at timestep ts.
%
% where Q is number of samples (or series) and TS is the number of timesteps.
%#ok<*RPMT0>

% ===== NEURAL NETWORK CONSTANTS =====
% Input 1
x1_step1.xoffset = [0.050000406938125;0.996004];
x1_step1.gain = [2.11415687495088;0.436973863575024];
x1_step1.ymin = -1;

% Layer 1
b1 = [2.2289609052132517419;-3.1035389316615029642;-2.4032063158139913561;-1.3370315150599720866;-
1.8948250242018953582;-1.2011816800690557905;1.7940585661691810238;4.0859595532787267302];
IW1_1 = [1.5246700524093336396 5.1721114124571014877;2.2432983260149210736 -
2.7817524756779241635;2.4374662270781768392 -3.8270275510585687329;3.1323617870437825417
2.1346370121054536462;-3.1311341950041424731 -0.24672115698921168181;-3.1134374575092405024 -
1.4010932770912736256;4.0936068244790835283 -1.7223509523009992161;3.4635092527491915249 -
1.6763568441034299994];

% Layer 2
b2 = -1.1329042900943480099;
LW2_1 = [-0.11371677726358328520 -0.19199587275602586489 -0.75291369483458392420 -1.3869374948451886898 -
0.57425742139245417039 -0.45319926513319874761 -0.13538171125155673558 -0.37797748282484211479];

% Output 1
y1_step1.ymin = -1;
y1_step1.gain = 0.0227272727272727;
y1_step1.xoffset = 1;

% ===== SIMULATION ========
% Format Input Arguments
isCellX = iscell(X);
if ~isCellX
  X = {X};
end

% Dimensions
TS = size(X,2); % timesteps
if ~isempty(X)
  Q = size(X{1},1); % samples/series
```

```
else
    Q = 0;
end

% Allocate Outputs
Y = cell(1,TS);

% Time loop
for ts=1:TS

    % Input 1
    X{1,ts} = X{1,ts}';
    Xp1 = mapminmax_apply(X{1,ts},x1_step1);

    % Layer 1
    a1 = tansig_apply(repmat(b1,1,Q) + IW1_1*Xp1);

    % Layer 2
    a2 = repmat(b2,1,Q) + LW2_1*a1;

    % Output 1
    Y{1,ts} = mapminmax_reverse(a2,y1_step1);
    Y{1,ts} = Y{1,ts}';
end

% Final Delay States
Xf = cell(1,0);
Af = cell(2,0);

% Format Output Arguments
if ~isCellX
    Y = cell2mat(Y);
end
end

% ===== MODULE FUNCTIONS ========
% Map Minimum and Maximum Input Processing Function
function y = mapminmax_apply(x,settings)
y = bsxfun(@minus,x,settings.xoffset);
y = bsxfun(@times,y,settings.gain);
y = bsxfun(@plus,y,settings.ymin);
end

% Sigmoid Symmetric Transfer Function
function a = tansig_apply(n,~)
a = 2 ./ (1 + exp(-2*n)) - 1;
end

% Map Minimum and Maximum Output Reverse-Processing Function
function x = mapminmax_reverse(y,settings)
x = bsxfun(@minus,y,settings.ymin);
x = bsxfun(@rdivide,x,settings.gain);
x = bsxfun(@plus,x,settings.xoffset);
end
```

Artificial neural network 2:

```
function [Y,Xf,Af] = myNeuralNetworkFunction(X,~,~)
%MYNEURALNETWORKFUNCTION neural network simulation function.
```

```
%
% Auto-generated by MATLAB, 04-Jan-2020 10:52:43.
%
% [Y] = myNeuralNetworkFunction(X,~,~) takes these arguments:
%
%   X = 1xTS cell, 1 inputs over TS timesteps
%   Each X{1,ts} = Qx2 matrix, input #1 at timestep ts.
%
% and returns:
%   Y = 1xTS cell of 1 outputs over TS timesteps.
%   Each Y{1,ts} = Qx1 matrix, output #1 at timestep ts.
%
% where Q is number of samples (or series) and TS is the number of timesteps.
%#ok<*RPMT0>

% ===== NEURAL NETWORK CONSTANTS =====
% Input 1
x1_step1.xoffset = [0.050000406938125;0.996004];
x1_step1.gain = [2.11415687495088;0.436973863575024];
x1_step1.ymin = -1;

% Layer 1
b1 = [4.0720549829091803318;-1.9901863782187132479;1.2741853088864916188;0.48409088401054189976;-
1.3238921741478488769;2.6066651740196475906;3.4522825126879737034];
IW1_1 = [-0.66762418035925219506 4.5089579055097024352;3.8318957794625507773 -1.372323390211348082;-
3.1082223119729324168 0.82346883613525134749;2.9325611819771153499 2.2449112026279811616;-
2.4505337736688246331 -2.4745878771559213227;1.3023489090947997227 -
3.4947301477898240485;0.074555722429619220026 3.7975684523068098386];

% Layer 2
b2 = -0.52965676121154148248;
LW2_1 = [0.28011400488540216802 -0.32310405323953583778 -0.47415167902349325546 -
0.66269548370648201363 -0.34778687799363616762 0.0020382154885661973864 0.29705647528451784023];

% Output 1
y1_step1.ymin = -1;
y1_step1.gain = 0.0227272727272727;
y1_step1.xoffset = 1;

% ===== SIMULATION ========
% Format Input Arguments
isCellX = iscell(X);
if ~isCellX
    X = {X};
end

% Dimensions
TS = size(X,2); % timesteps
if ~isempty(X)
    Q = size(X{1},1); % samples/series
else
    Q = 0;
end

% Allocate Outputs
Y = cell(1,TS);

% Time loop
for ts=1:TS
```

```
    % Input 1
    X{1,ts} = X{1,ts}';
    Xp1 = mapminmax_apply(X{1,ts},x1_step1);

    % Layer 1
    a1 = tansig_apply(repmat(b1,1,Q) + IW1_1*Xp1);

    % Layer 2
    a2 = repmat(b2,1,Q) + LW2_1*a1;

    % Output 1
    Y{1,ts} = mapminmax_reverse(a2,y1_step1);
    Y{1,ts} = Y{1,ts}';
end

% Final Delay States
Xf = cell(1,0);
Af = cell(2,0);

% Format Output Arguments
if ~isCellX
    Y = cell2mat(Y);
end
end

% ===== MODULE FUNCTIONS ========
% Map Minimum and Maximum Input Processing Function
function y = mapminmax_apply(x,settings)
y = bsxfun(@minus,x,settings.xoffset);
y = bsxfun(@times,y,settings.gain);
y = bsxfun(@plus,y,settings.ymin);
end

% Sigmoid Symmetric Transfer Function
function a = tansig_apply(n,~)
a = 2 ./ (1 + exp(-2*n)) - 1;
end

% Map Minimum and Maximum Output Reverse-Processing Function
function x = mapminmax_reverse(y,settings)
x = bsxfun(@minus,y,settings.ymin);
x = bsxfun(@rdivide,x,settings.gain);
x = bsxfun(@plus,x,settings.xoffset);
end
```

Artificial neural network 3:

```
function [Y,Xf,Af] = myNeuralNetworkFunction(X,~,~)
%MYNEURALNETWORKFUNCTION neural network simulation function.
%
% Auto-generated by MATLAB, 04-Jan-2020 11:23:27.
%
% [Y] = myNeuralNetworkFunction(X,~,~) takes these arguments:
%
%   X = 1xTS cell, 1 inputs over TS timesteps
%   Each X{1,ts} = Qx2 matrix, input #1 at timestep ts.
%
% and returns:
```

```
%   Y = 1xTS cell of 1 outputs over TS timesteps.
%   Each Y{1,ts} = Qx1 matrix, output #1 at timestep ts.
%
% where Q is number of samples (or series) and TS is the number of timesteps.

%#ok<*RPMT0>

% ===== NEURAL NETWORK CONSTANTS =====
% Input 1
x1_step1.xoffset = [0.050000406938125;0.996004];
x1_step1.gain = [2.11415687495088;0.436973863575024];
x1_step1.ymin = -1;

% Layer 1
b1 = [-5.0797644800616126304;-5.1451108110727599154;6.4376376660823444453;-
4.4372002617230981159;1.3388251032720461797;0.93351720648673375269;-1.1044086274556381699;-
1.3994336270452454318;-1.2052177941945643l8;3.3168220765667153493;-2.9207852579476485388;-
3.9232142833533147908;-5.123947479344723277];
IW1_1 = [2.3185182081560533618 4.4523485073456248173;2.6869045207435471312 -3.8251464250755429219;-
5.3230754451703710117 -0.53812229285161072845;3.6838686427395344047 2.8191338798274094835;-
2.8982313282519047704 5.6523755998916387355;-3.0270134623041244915 -3.6686030470949102522;-
0.36648691515664327811 6.1148312392971364559;-1.9148851141609153359 3.8398733357143979283;-
0.62049712311309301871 -5.3064628817515817616;4.2579204739726366213 -2.4113952334209534101;-
5.3544777698180618941 -1.9791978901140792058;-4.8880657496066888257 0.74980944707615315714;-
2.7275869252238988949 -4.2265053134123817813];

% Layer 2
b2 = -1.2558061025047497772;
LW2_1 = [1.7135300339909140 81 -0.57062839491819239424 0.67799773882488756 36 -0.60783989051065301368 -
0.6437839567872610802 0.77250051073480807684 1.2072000784083731784 -1.0631356977375712169 -
0.96597408259568162681 1.5267769264970960652 0.58442868672967573929 1.0163009100141400243 -
1.0736801919297125352];

% Output 1
y1_step1.ymin = -1;
y1_step1.gain = 0.0227272727272727;
y1_step1.xoffset = 1;

% ===== SIMULATION ========
% Format Input Arguments
isCellX = iscell(X);
if ~isCellX
    X = {X};
end

% Dimensions
TS = size(X,2); % timesteps
if ~isempty(X)
    Q = size(X{1},1); % samples/series
else
    Q = 0;
end

% Allocate Outputs
Y = cell(1,TS);

% Time loop
for ts=1:TS
```

```
    % Input 1
    X{1,ts} = X{1,ts}';
    Xp1 = mapminmax_apply(X{1,ts},x1_step1);

    % Layer 1
    a1 = tansig_apply(repmat(b1,1,Q) + IW1_1*Xp1);

    % Layer 2
    a2 = repmat(b2,1,Q) + LW2_1*a1;

    % Output 1
    Y{1,ts} = mapminmax_reverse(a2,y1_step1);
    Y{1,ts} = Y{1,ts}';
end

% Final Delay States
Xf = cell(1,0);
Af = cell(2,0);

% Format Output Arguments
if ~isCellX
    Y = cell2mat(Y);
end
end

% ===== MODULE FUNCTIONS ========
% Map Minimum and Maximum Input Processing Function
function y = mapminmax_apply(x,settings)
y = bsxfun(@minus,x,settings.xoffset);
y = bsxfun(@times,y,settings.gain);
y = bsxfun(@plus,y,settings.ymin);
end

% Sigmoid Symmetric Transfer Function
function a = tansig_apply(n,~)
a = 2 ./ (1 + exp(-2*n)) - 1;
end

% Map Minimum and Maximum Output Reverse-Processing Function
function x = mapminmax_reverse(y,settings)
x = bsxfun(@minus,y,settings.ymin);
x = bsxfun(@rdivide,x,settings.gain);
x = bsxfun(@plus,x,settings.xoffset);
end
```
